# RNA sequencing of synaptic and cytoplasmic Upf1-bound transcripts supports contribution of nonsense-mediated decay to epileptogenesis

**DOI:** 10.1038/srep41517

**Published:** 2017-01-27

**Authors:** Claire M. Mooney, Eva M. Jimenez-Mateos, Tobias Engel, Catherine Mooney, Mairead Diviney, Morten T. Venø, Jørgen Kjems, Michael A. Farrell, Donncha F. O’Brien, Norman Delanty, David C. Henshall

**Affiliations:** 1 Department of Physiology & Medical Physics, Royal College of Surgeons in Ireland, Dublin, Ireland; 2 Department of Molecular Biology and Genetics and Center for DNA Nanotechnology and Interdisciplinary Nanoscience Center (iNANO), Aarhus University, Aarhus C, Denmark; 3 Beaumont hospital, Beaumont, Dublin, Ireland

## Abstract

The nonsense mediated decay (NMD) pathway is a critical surveillance mechanism for identifying aberrant mRNA transcripts. It is unknown, however, whether the NMD system is affected by seizures *in vivo* and whether changes confer beneficial or maladaptive responses that influence long-term outcomes such the network alterations that produce spontaneous recurrent seizures. Here we explored the responses of the NMD pathway to prolonged seizures (status epilepticus) and investigated the effects of NMD inhibition on epilepsy in mice. Status epilepticus led to increased protein levels of Up-frameshift suppressor 1 homolog (Upf1) within the mouse hippocampus. Upf1 protein levels were also higher in resected hippocampus from patients with intractable temporal lobe epilepsy. Immunoprecipitation of Upf1-bound RNA from the cytoplasmic and synaptosomal compartments followed by RNA sequencing identified unique populations of NMD-associated transcripts and altered levels after status epilepticus, including known substrates such as *Arc* as well as novel targets including *Inhba* and *Npas4*. Finally, long-term video-EEG recordings determined that pharmacologic interference in the NMD pathway after status epilepticus reduced the later occurrence of spontaneous seizures in mice. These findings suggest compartment-specific recruitment and differential loading of transcripts by NMD pathway components may contribute to the process of epileptogenesis.

Important check-points exist on the journey from gene transcription to translation that support surveillance of the quality of mRNA transcripts in order to prevent translation of aberrant transcripts which could lead to the production of mutated or harmful proteins^1^. The nonsense mediated decay (NMD) pathway is a key mechanism of RNA surveillance that targets both aberrant and physiologically occuring transcripts for decay^2^,^3^,^4^,^5^. The NMD system comprises a multi-component complex containing various evoluntionarily conserved proteins. Up-frameshift suppressor 1 homolog (Upf1) forms a complex with other NMD proteins including Upf2 and Upf3b among others, and phosphorylation of Upf1 initiates the decay process^4^. It is estimated that approximately 2–10% of mammalian mRNAs are subject to NMD^6^,^7^,^8^. The classic mammalian substrates for NMD are mRNA transcripts harbouring a premature termination codon (PTC) on an internal exon. Targeting of transcripts containing nonsense and frameshift mutations by the NMD pathway prevents production of potentially pathogenic truncated or aberrant proteins and mutation of genes encoding NMD proteins is associated with several rare and often fatal diseases^9^. However, it is now understood that NMD also targets physiologically occurring transcripts such as those with open reading frames located upstream of the main coding region, with introns in their 3′untranslated region (UTR) and long 3′ UTRs^5^,^10^.

The brain displays one of the highest rates of NMD activity among tissues^11^. NMD function is important in control of synaptic plasticity by regulating the local translation of transcripts within dendrites including the immediate early gene *Arc*^12^. During nervous system development NMD shapes the growth and trajectory of axons^13^ and NMD pathway components influence neural progenitor cell proliferation and neuronal differentiation^14^. Alternative splicing of certain transcripts results in exon-skipping or inclusion allows these mRNAs, which would normally be degraded by the NMD system, to by-pass the pathway and produce functional mRNAs required for neuronal maturation and functioning^15^,^16^. Loss-of-function mutations in NMD proteins result in a variety of neurological disorders including intellectual disability, autism and schizophrenia^17^,^18^.

Nonsense mutations have been described in γ-amino butyric acid (GABA) receptors and synaptic proteins which have been implicated in the human epilepsies and experimentally verified to be targeted by the NMD system^19^,^20^,^21^,^22^,^23^. Whether the NMD system is involved in the pathophysiologic changes associated with the development of epilepsy – epileptogenesis - has not been investigated. Profiling studies indicate that between ~300–1500 mRNA transcripts are differentially regulated in the hippocampus after status epilepticus and other epilepsy-provoking insults^24^,^25^,^26^,^27^, some of which are also regulated in human epilepsy^28^,^29^. Their encoded proteins are thought to influence adaptive and maladaptive processes including synaptic and axonal reorganisation, neurotransmitter signalling pathways, metabolism, neurogenesis, inflammation and reorganisation of the extracellular matrix^30^. It is likely that surveillance of injury-induced transcripts by the NMD system has an important influence on epileptogenesis. Here we investigated the expression of NMD components in a mouse model of status epilepticus. We evaluated changes to NMD-associated transcripts provoked by status epilepticus by immunoprecipitating RNA from Upf1 (RIP-seq) and we tested the effects of a small molecule inhibitor of the NMD system on the occurrence of spontaneous seizures.

## Materials and Methods

### Animal model of status epilepticus

All procedures were performed in accordance with the guidelines of the European Communities Council Directives (86/609/EU and 2010/63/EU) and were reviewed and approved by the Research Ethics Committee of the Royal College of Surgeons in Ireland (REC #205, 842) under license from the Department of Health and Health Products Regulatory Authority, Dublin, Ireland (AE19127/P001). Male, C57BL/6 mice (Harlan, 6–8 weeks old) were used in all studies. Intraamygdala kainic acid (KA)-induced status epilepticus was induced as previously described^31^. Briefly, mice were anaesthetised using isoflurane and placed in a stereotaxic frame, affixed with skull-mounted recording electrodes. A craniectomy was performed and a guide cannula placed on the skull over the right amygdala. The assembly was cemented in place and then mice were placed in a warmed recovery chamber. Next, KA (0.3 μg in 0.2 μl volume) was administered to freely-moving mice via microinjection through the guide cannula into the right amygdala. Seizures began within 5–10 minutes and continued with increasing severity until becoming continuous after approximately 30 minutes. Lorazepam (8 mg/kg, intraperitoneal) was administered 40 minutes after KA to reduce morbidity and mortality and restrict the extent of hippocampal damage.

### NMD inhibitor treatment

NMD inhibition was achieved using a small molecule inhibitor, NMDI14^32^. For this, an additional craniectomy was performed at the time of affixing electrodes and the guide cannula for intraamygdala injection. NMDI14 (2 μl, 674 μM) was injected 1, 4 and 24 hours after seizure termination by lorazepam into the ventricle in order to achieve an approximate final ventricular concentration of 50 μM NMDI14 based on a dose that achieved maximum reduction in p-Upf1 (see results).

### EEG monitoring

Acute EEG during status epilepticus in mice was recorded using a Grass Comet XL digital EEG. Seizure activity was defined as high amplitude high frequency discharges (HAHFDs; two-fold increase in amplitude over baseline and frequency >1 Hz lasting ≥5 seconds) recorded within the 40 minute period following KA administration. For long-term epilepsy monitoring studies, continuous EEG and video recordings were performed using implantable EEG telemetry devices (Data Systems International)^31^. Transmitters (model F20-EET) which record bilateral EEG from the skull were implanted in a subcutaneous pocket at the time of cannula placement. The behaviour of the animals was recorded using a video camera placed next to the cage. Continuous video-EEG data were acquired for each animal, beginning on the first day of spontaneous seizures in this model (day 3 after status epilepticus) until the end of the study (day 14)^31^. For two animals in the control group, telemetry devices failed before the end of the study (day 10 and day 13) and spontaneous seizures occurring after this time were scored by video-only. Spontaneous seizures were defined as high frequency (>5 Hz), high amplitude (>2 times baseline) polyspike discharges of ≥5 s duration. For detection of seizures on the days when the EEG data was missing, a seizure was confirmed by the following behaviours: sudden immobility and freezing, forelimb and or tail extension, rigid posture, repetitive uncontrollable movements, head bobbing, rearing and falling, and severe tonic–clonic seizures^33^.

### Human brain samples

This study was approved by the Ethics (Medical Research) Committee of Beaumont Hospital, Dublin (#05/18, #13/75) and written informed consent was obtained from all patients. All human-related study methods were performed in accordance with all relevant legislative requirements and the research was conducted in accordance with the ethical principles outlined in the Declaration of Helsinki and Good Clinical Practice. Patients (*n* = 6) were referred for surgical resection of the temporal lobe for the treatment of intractable temporal lobe epilepsy (TLE). After temporal lobe resection, the hippocampus was divided, with one piece frozen in liquid nitrogen and stored at −80 °C. The remaining bloc was processed for routine pathological analysis and samples were assessed for the presence of hippocampal sclerosis and other pathological changes. Control (autopsy) hippocampi (*n* = 6) were obtained from individuals with no history of neurological disease from the Brain and Tissue Bank for Developmental Disorders at the University of Maryland, Baltimore, MD, USA. Sex ratios for each group were 2 M:4 F for TLE patients and 3 M:3 F for autopsy controls. Average ages for each group were: 33.5 years (range 17–54) for TLE patients and 42.3 years (range 37–50) for controls. The average post-mortem interval was 15 hours. Full details of control and patient pathology and clinical data can be found in Supplementary table S1.

### Western blotting

Western blotting was performed as described^34^. Briefly, extracted proteins were separated on SDS-PAGE gels and transferred to nitrocellulose membranes. Membranes were blocked for 1 h our with either 5% milk or 5% BSA, incubated with either Upf1 (Abcam ab109363), p-Upf1 (Millipore 07-1016), Upf2 (GeneTex GTX107694), Upf3b (Aviva Systems Biology ARP40998_T100), Tubulin (Sigma T6199), Actin (Sigma A5441), Synaptophysin (Sigma S5768), mGluR6/7 (Novus Biologicals NM120-15307), Porin (Calbiochem 529536) or Lamin A/C (Cell Signalling Technology 2032 S) overnight at 4 °C, and finally incubated with the horseradish peroxidase conjugated secondary antibodies (Cell Signalling Technology). Protein bands were visualized using SuperSignal^®^ West Pico Chemiluminescent Substrate (Millipore) and imaged using a Fuji-film LAS-3000. Band densities were analysed using ImageJ software. Protein levels were corrected to Actin, Tubulin or synaptophysin (synaptoneurosomes) loading controls.

### Synaptoneurosomes

Synaptoneurosomes (SN) were prepared from mouse hippocampi according to previous techniques^35^,^36^. For each SN preparation 2–6 hippocampi were combined. Hippocampi were homogenised in ice cold homogenising buffer (0.32 M sucrose, 1 mM ethylenediaminetetraacetic acid (EDTA), 1 mg/mL bovine serum albumin, 5 mM 4-(2-hydroxyethyl)piperazine-1-ethanesulfonic acid (HEPES) pH 7.4) using a Glass Teflon douncer (10 strokes at 600–650 rpm). The homogenate was then centrifuged at 3000 × g for 10 minutes at 4 °C. The supernatant containing the SN and cytoplasmic cellular fraction was decanted and centrifuged again at 14,000 × g for 12 minutes at 4 °C. The supernatant containing the cytoplasmic fraction was retained on ice while the SN-containing pellet was re-suspended in 110 μL of Krebs-Ringer buffer (0.14 M NaCl, 5 mM KCl, 5 mM glucose, 1 mM EDTA, 0.01 M HEPES pH 7.4). To this, 90 μL Percoll was added to make a final concentration of Percoll 45% v/v, tubes were inverted to mix and then centrifuged at 14,000 × g for 2 minutes at 4 °C. The enriched SN fraction was removed from the surface using a pipette, re-suspended in 1 mL of Krebs-Ringer buffer and centrifuged at 14,000 × g for 30 seconds. The SN pellet was then re-suspended in lysis buffer (0.15 M NaCl, 0.05 M Tris HCl pH 8.0, 1% NP-40 0.001 M EDTA pH 8.0). Protein content in each fraction was quantified using the micro BCA method (Protein Quantification kit, Pierce Scientific). Finally, after addition of 6X SDS loading buffer, the SN samples were heated at 95 °C for 10 minutes and either used immediately for western blotting or stored at −80 °C.

### Immunohistochemistry

Free-floating sections (30 μm) were obtained from mice perfused with 4% paraformaldehyde (PFA). Sections were incubated with 0.1% Triton-X/PBS for 15 minutes, after which 1 M glycine was added and incubated for 30 minutes. Sections were then washed in PBS and incubated with 1% Bovine Serum Albumin (BSA)/PBS for 1 hour to block unspecific binding of primary antibodies to the sections. Sections were then incubated with Upf1 (Abcam ab109363), AnkyrinB (NeuroMab 75–145) and/or Map2 (Sigma M9942) antibody overnight at 4 °C, washed three times for 5 minutes with PBS, incubated with fluorescently-labelled secondary antibodies Alexa-fluor 488 and Alexa-fluor 546 and nuclear marker Hoechst, washed with PBS and finally mounted on glass slides and coverslipped with Fluorsave (Millipore). Sections were imaged using a Leica DM400B fluorescent microscope or a Zeiss Axiovert 200 M confocal microscope.

### RIP-Seq

All buffers and reagents used in the preparation of samples for RNA-seq were RNAse free. Pools of hippocampi from control and SE (*n* = 6 each) mice were combined and synaptoneurosome and cytoplasmic fractions were isolated as described above. For these studies it was necessary to pool large numbers of hippocampi and therefore biological replicates were not attempted. Upf1 (Abcam ab109363) was immunoprecipitated from samples using Protein-G-conjugated Dynabeads (Thermo Fisher Scientific) and Upf1-bound RNA was extracted using TRIzol (Thermo Fisher Scientific). Ribosomal RNA was removed from the co-precipitated RNA using the Ribo-Zero Magnetic Kit (Illumina) and the RNA library was constructed using the ScriptSeqTM v2 RNA-Seq Library Preparation Kit (Illumina) according to the manufacturer’s instructions. 3′Adapter sequences were removed and the quality of sequenced reads were first checked using FASTqc software^37^. Reads were then mapped to the mouse genome (Dec. 2011 (GRCm38/mm10) assembly) using Bowtie, analysed using TopHat and Cufflinks free open source software^38^,^39^. FPKM cut-off for gene presence was ≥1.

### Bioinformatic analysis of RIP-seq data

Transcripts identified by RIP-seq were categorised based on their regulation. Unregulated transcripts were sorted into 3 groups: those present in both the cytoplasm and synaptoneurosomes, those only found in synaptoneurosomes and those found exclusively in the cytoplasm. Regulated genes were divided into 4 groups: Genes expressed exclusively in control SNs or with a log2-fold decrease ≤−1.5, genes expressed exclusively in SE SNs or with a log2-fold increase ≥−1.5, genes expressed exclusively in control cytoplasm or with a log2-fold decrease ≤−1.5, genes expressed exclusively in SE cytoplasm or with a log2-fold increase ≥−1.5. Online open source software Panther^40^ was used for gene ontology (GO) and pathway analysis of the 7 gene lists. Significant over- and under-represented GO terms and pathways were identified by p < 0.05.

### RNA extraction and real-time PCR

Total RNA was extracted using the Trizol method^34^. For analysis of *Arc, FosB, Inhba, Npas4, Pcdh8, Tll1* and *Slc1a2* transcripts, cDNA was produced from the total Upf1-precipitated RNA by reverse transcription using Superscript II Reverse Transcriptase enzyme (Invitrogen). Quantitative real-time PCR was performed using a LightCycler 1.5 (Roche Diagnostics) and QuantiTech SYBR Green PCR kit (Qiagen) as per the manufacturer’s instructions and 1.25 μM of primer pair was used. *Arc* forward: AGCAGCAGACCTGACATCCT, reverse: GTGATGCCCTTTCCAGACAT; *FosB* forward: AGGAACCAGCTACTCAACCC, reverse: AAGTCGATCTGTCAGCTCCC; *Inhba* forward: CATCACCTTTGCCGAGTCAG, reverse: AGACGGATGGTGACTTTGGT; *Npas4* forward: TGAAGACATTGTGGCAGCAC, reverse: TGGTCAGCAGGGTCAATGAT; *Pcdh8* forward: ATCGGAACCCTTGCAGAAGA, reverse: CTGACAACATCGAAGGCCAG; *S18* forward: AAGAGGGCTGGAGAACTCA, reverse: GCAGCTTGTTGTCTAGACCG; *Slc1a2* forward: GCCTGCTTGATTTGTGGGAA, reverse: AGTTCCCAGAGCAGTGATCC; *Tll1* forward: CGCCAAGCCAGTACAGAATC, reverse: CACTTCAGGTATGTCAGCGC). Data were normalized to expression of S18 RNA.

### Data analysis and statistics

Data are presented as means ± standard error of the mean (SEM). Data were analysed using ANOVA with either Dunnet’s post-hoc test when comparing all groups to just one group and Bonferroni’s post-hoc analysis otherwise. Student’s t-test for two-group comparison and Mann–Whitney test for qPCR validation of RNA sequencing results. Significance was accepted at p < 0.05. For RIP-seq analysis Bowtie open access software was used^41^. Briefly, identification of raw reads was carried out using Tophat. These reads were then aligned to the mouse genome (USCS Genome browser Dec2011 GRCm38/mm10) using cufflinks. Using the cufflinks output FPKM values, differences between samples were calculated using Cuffdiff which calculates the log_2_ fold-change in a given transcript and significant p values (both raw and corrected for multiple testing).

### Ethics approval and consent to participate

Animal studies were approved by the Research Ethics Committee of the Royal College of Surgeons in Ireland (REC) under license from the Department of Health and Health Products Regulatory Authority, Dublin, Ireland. Human tissue studies were approved by the Ethics (Medical Research) Committee of Beaumont Hospital, Dublin (05/18). Written informed consent was obtained from all epilepsy patients.

## Results

### Status epilepticus results in selective alterations to hippocampal levels of NMD proteins

**Figure 1:**
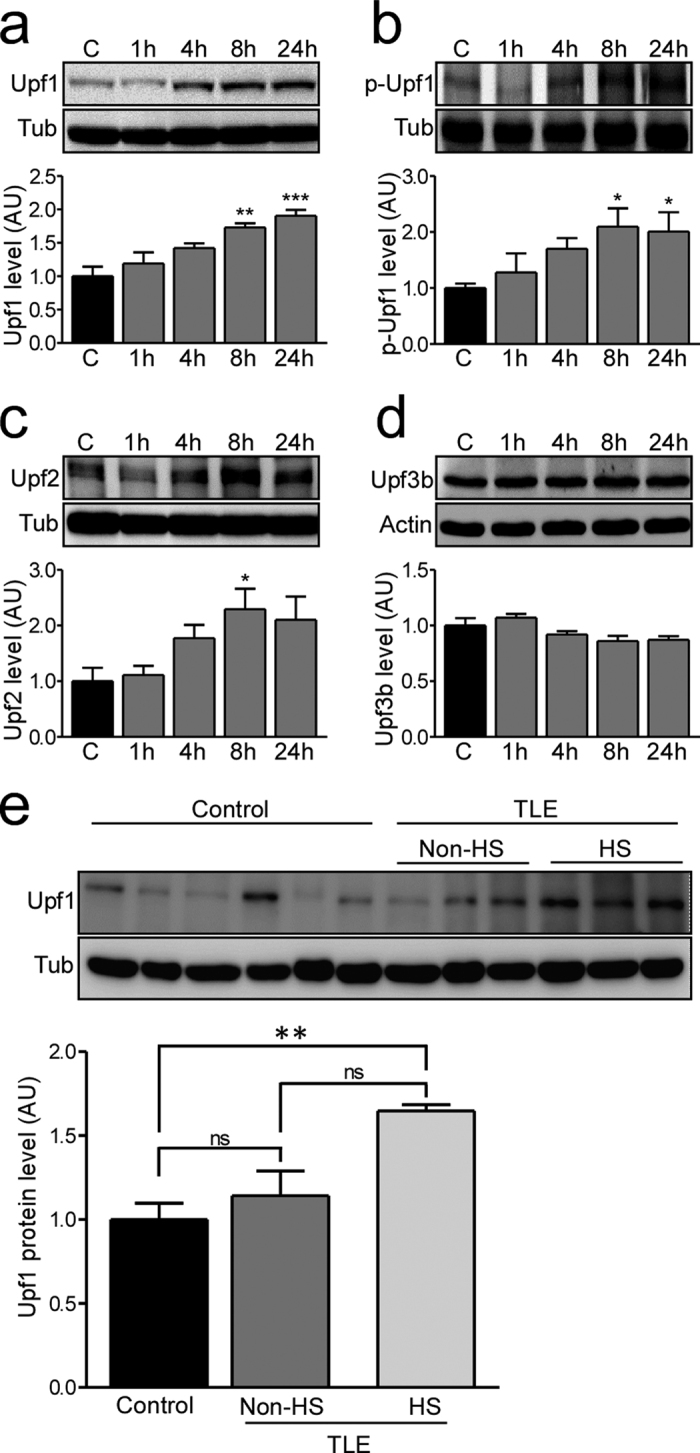
NMD proteins are increased after SE in mice and Upf1 is increased in human TLE samples. (**a–d**) Protein levels of Upf1, phosho-Upf1 (p-Upf1), Upf2 and Upf3b in the ipsilateral hippocampus in control (C) mice and at 1, 4, 8 and 24 hours (h) after SE were analysed by western blot and semi-quantified. (**a**) Upf1 levels significantly increased 8 and 24 h after SE (n = 4/group; ANOVA, Dunnett’s posthoc test ***p < 0.001, **p < 0.01). (**b**) p-Upf1 levels significantly increased 8 and 24 h after SE (n = 5/group; ANOVA, Dunnett’s posthoc test *p < 0.05 compared to control. (**c**) Upf2 levels were increased 8 h after SE (n = 4/group; ANOVA, Dunnett’s posthoc test *p < 0.05 compared to control). (**d**) Upf3b levels did not change after SE (n = 4/group; ANOVA, Dunnett’s posthoc test p = 0.88). (**e**) Upf1 protein levels were significantly higher in TLE patients with hippocampal sclerosis (HS) compared to post-mortem controls and TLE patients without HS (Controls n = 6; TLE without HS n = 3, TLE with HS n = 3; ANOVA with Bonferroni post-hoc test comparing all columns; p = 0.0072). Representative blots have been cropped to reduce unnecessary area.

Multiple components of the NMD system were expressed in the hippocampus of control mice, including Upf1, Upf2 and Upf3b (Fig. 1). To determine if status epilepticus had any effect on the expression of NMD proteins we compared levels of these proteins and phospho-Upf1, the active form, 1, 4, 8 and 24 hours after status epilepticus induced by intraamygdala KA in mice. In this model, prolonged seizures are triggered from the amygdala that propagate and recruit the hippocampus leading to unilateral hippocampal damage^31^,^35^.

Western blot analysis revealed an increase in Upf1 and p-Upf1 at 8 and 24 hours (Fig. 1a,b) and Upf2 at 8 hours (Fig. 1c) after status epilepticus in the model. In contrast, the levels of Upf3b remained unchanged after status epileticus (Fig. 1d). To explore the translational relevance of these findings we investigated Upf1 expression in human hippocampus. For these studies we obtained a collection of resected hippocampi from patients with intractable TLE who had undergone surgical resection. This included tissue samples both with and without hippocampal sclerosis, a hallmark pathologic finding comprising selective neuronal loss and gliosis (Supplementary table S1). Upf1 was detected at varying levels in autopsy control hippocampi (Fig. 1e). Comparison of Upf1 levels in these samples determined that Upf1 levels were higher in TLE patient samples compared to post-mortem controls (Fig. 1e; P = 0.037)). This was not an artefact of autopsy delay (Supplementary figure S1). There was no difference in the levels of Upf1 between patients with and without hippocampal sclerosis or patients without hippocampal sclerosis and postmortem controls (Fig. 1e).

### Increased synaptic localisation of Upf1 after status epilepticus in mice

**Figure 2:**
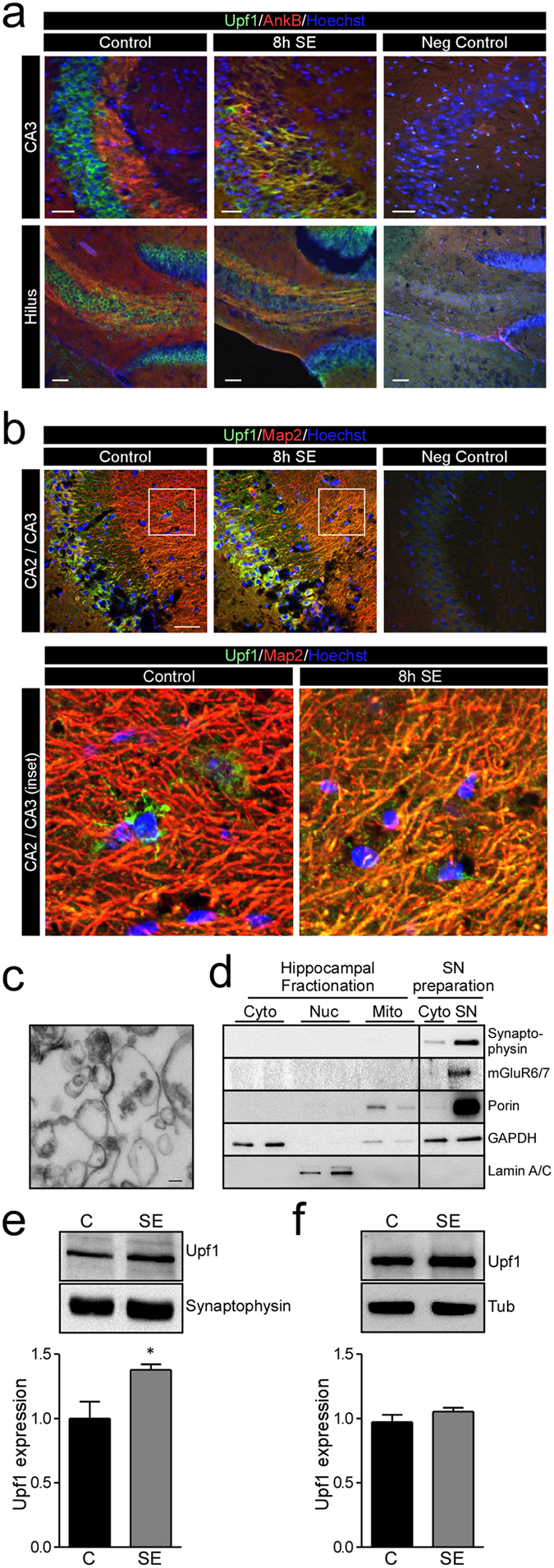
Hippocampal re-localization of Upf1 after SE in mice. (**a, b**) Representative photomicrographs of mouse hippocampal sections stained with antibodies against Upf1 (green), dendrite marker protein Map2 (red) or axon marker protein AnkyrinB (AnkB, red) and Hoechst (blue). Negative control sections were prepared with no primary antibodies. Images show increased Upf1 colocalisation with AnkB in the CA3 and hilus subfields (**a**) and increased colocalisation with Map2 in the CA2/CA3 regions (**b**) 8 h after SE. (**c**) Mouse synaptoneurosomes (SN) were prepared and synaptic enrichment was confirmed using electron microscopy by the presence of morphological features of synaptoneurosome including a dark post-synaptic density between a synapsome and synaptoneurosome. (**d**) Western blot analysis confirming enrichment of synaptic proteins synaptophysin and mGluR6/7 and mitochondrial protein porin. (**e**) Western blot analysis of Upf1 levels show Upf1 was increased in synaptoneurosomes 8 h after SE in mice (Control n = 4, SE n = 5; student’s t-test p = 0.019). (**f**) SE did not significantly alter Upf1 levels in the corresponding cytoplasmic fraction (Control n = 4, SE n = 5; student’s t-test p = 0.2130). Representative blots have been cropped to reduce unnecessary area.

Previous studies have indicated that NMD is active at distal sites from the nucleus during neuronal development and after neuronal activity^12^,^13^,^17^. Upf1 staining in control sections of the mouse hippocampus had a mainly neuronal distribution (Fig. 2). To determine whether status epilepticus drives changes in Upf1 localization we double-stained brain sections with specific antibodies against Upf1 and either the axonal marker AnkyrinB or the dendritic marker Map2. Immunofluorescent images revealed enhanced co-localisation of Upf1 with AnkyrinB in areas of damage after status epilepticus, including the CA3 subfield and hilar regions of the hippocampus (Fig. 2a). Upf1 also co-localized with Map2, a marker of dendrites, in the hippocampus after status epilepticus, particularly around area CA2 (Fig. 2b). Specificity of the staining was confirmed by ommission of the primary antibody (Fig. 2b).

To support these observations we generated synaptoneurosomes (SN) from the hippocampus of control and seizure mice. These are isolated portions of neurons containing resealed presynaptic structures (synaptosomes) together with attached sealed postsynaptic entities (neurosomes)^42^. The purity of the SN preparation was confirmed by electron microscopy (Fig. 2c) and immunoblotting with specific marker proteins of various cellular compartments (Fig. 2d). Western blot analysis of SN samples showed increased levels of Upf1 after status epilepticus (Fig. 2e). In contrast, Upf1 levels did not change after status epilepticus within samples from the cytoplasm (Fig. 2f).

### RIP-seq analysis of Upf1-bound transcripts after status epilepticus

**Figure 3:**
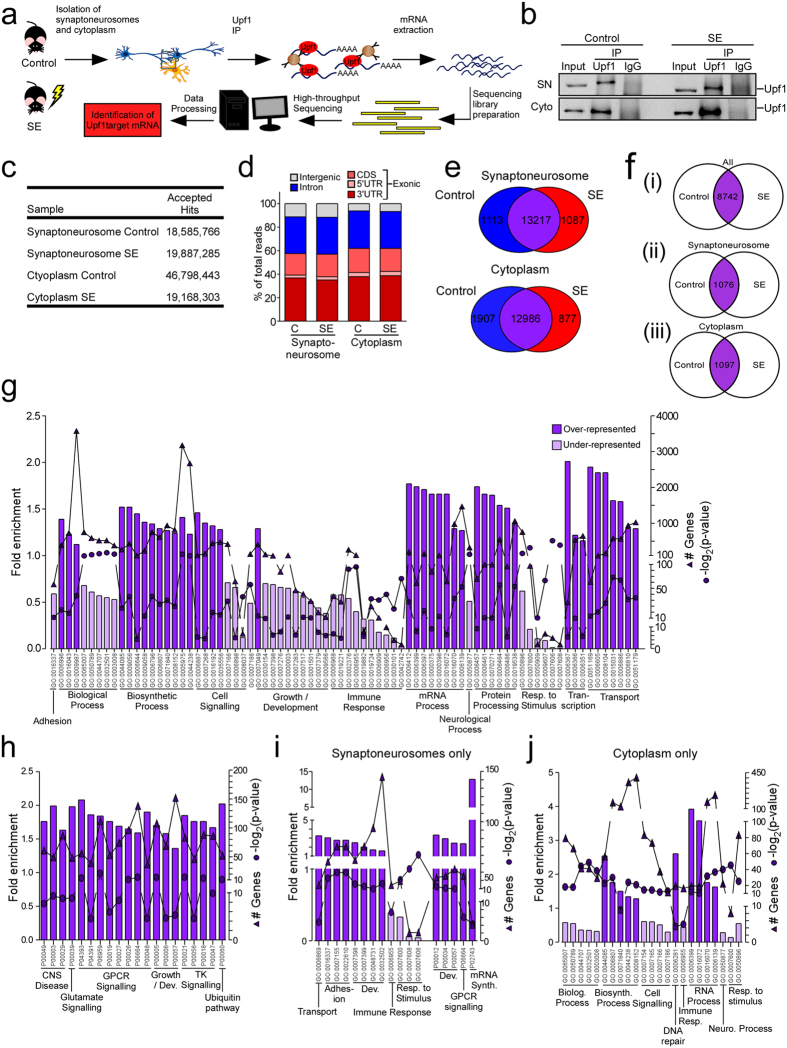
RIP- seq analysis of Upf1-bound transcripts in synaptoneurosomes and cytoplasm after SE. (**a**) Schematic of RIP-seq experimental design. Original drawing by author Claire Mooney. (**b**) Immunoblot showing Upf1 pull-down in a separate experiment in samples from synaptoneurosome (SN) and cytoplasm compartments generated from hippocampi from control and SE (8 h) mice. Representative blots have been cropped to reduce unnecessary area. (**c**) Raw reads in each sample. (**d**) Distribution of raw reads from control and SE samples of cytoplasm and synpatoneurosome fractions mapped to exonic gene regions including the 3′UTR, 5′UTR and coding sequence (CDS), intronic gene regions and intergenic regions. (**e**) The majority of transcripts were expressed in control and SE samples in synaptoneurosomes and cytoplasm while ~15% of transcripts were exclusive to either control or SE in synaptoneurosomes and cytoplasm. (**f**) Venn diagrams indicate the number of unchanged/unregulated Upf1-bound mRNAs expressed in either both SN and cytoplasm factions (i) or exclusively found in either SNs (ii) or cytoplasm (iii). (**g**) GO analysis of the gene lists from F(i) indicates over- and under-represented GO terms and pathways associated with genes expressed in both SNs and cytoplasm. (**h**) Pathways associated with genes expressed in both the SN and cytoplasm. (**i**) Biological processes and pathways associated with genes only found in SNs (F(ii) group). (**j**) Biological processes related to genes only found in cytoplasm (F(iii) group).

We next sought to determine whether there were changes in Upf1-targeting of mRNA transcripts after status epilepticus. To this end, we used RNA-immunoprecipitation sequencing (RIP-seq)^43^. We performed RIP-seq analysis of Upf1 from control and status epilepticus samples (8 h) for both SN and cytoplasmic Upf1-bound transcripts. The workflow for Upf1 RIP-seq is presented in Fig. 3a and we confirmed Upf1 pull-downs by western blotting (Fig. 3b).

The number of reads (hits) from RIP-seq experiments were between 18–47 × 10^6^ (Fig. 3c). We then analysed the gene regions that these reads mapped to, which showed the 3′UTR gene region was highly enriched in all samples accounting for between 35.1%–38.7% (compared to 8% for another RNA binding protein TDP-43^44^) of all reads across each sample (Fig. 3d) suggesting Upf1 enrichment on 3′UTRs. This was also confirmed when we examined the distribution of Upf1-binding to the some of the mRNA targets. A total of 15,417 and 15,770 transcripts were identified in SN and cytoplasmic fractions, respectively (Fig. 3e).

Since the targets of compartmentalized Upf1 in the normal hippocampus are unknown we next queried the type of transcripts that are targeted by Upf1 in SNs and cytoplasm. GO analyses of the set of transcripts that were unregulated in both SN and cytoplasm and exclusively in either SNs or cytoplasm of control hippocampus are shown in Fig. 3f (and see Supplementary tables S2–4). Biological processes relating to biosynthetic mechanisms, mRNA processes, protein processing, transcription and transport and pathways relating to G protein couple receptors (GPCR), tyrosine kinase and glutamate signalling, CNS diseases, growth/development and ubiquitination were over-represented in the cytoplasm and SN combined (Fig. 3g–h). Surprisingly, the GO term relating to neurological process was under-represented in this group (Fig. 3g). Over-represented biological processes such as adhesion and development and pathways relating to mRNA synthesis were unique to SNs (Fig. 3i) while genes related to DNA repair were unique to the cytoplasm (Fig. 3j). GO terms associated with immune and stimulus responses were under-represented in control and all datasets, respectively (Fig. 3g,i,j). We compared the over-represented GO terms and pathways identified from our data with those generated using available lists of NMD targets from other studies in mouse and human tissue^8^,^45^,^46^,^47^. This analysis showed some overlap between mouse and human datasets with a number of novel GO terms identified in our data (Supplementary figure S2).

**Figure 4:**
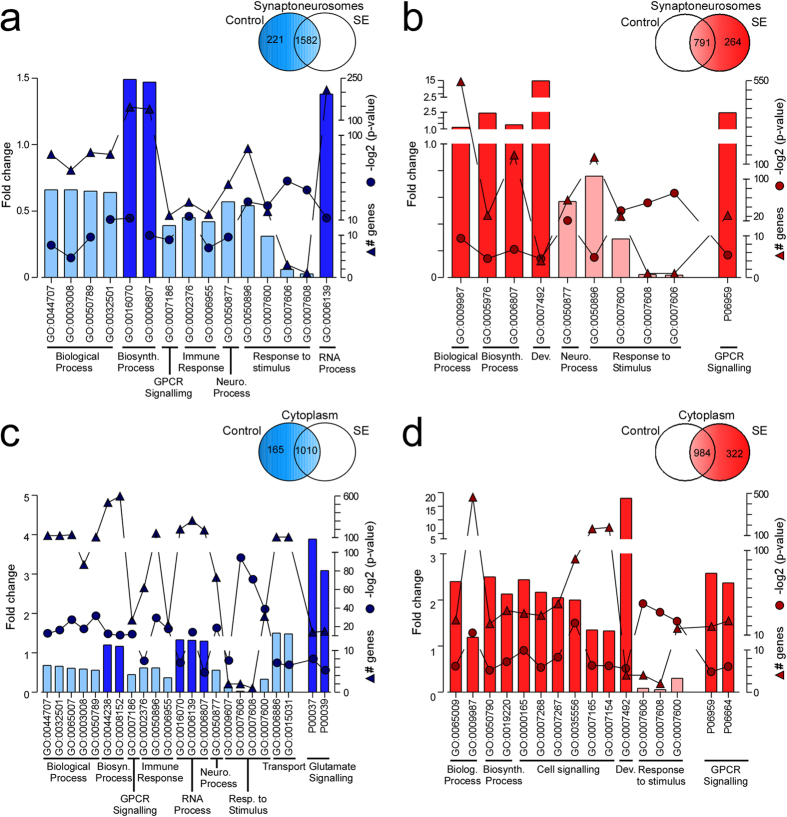
Bioinformatics analysis of Upf1-bound transcripts with altered expression after SE. Upf1-bound transcripts whose log2-fold expression was decreased by 1.5 fold or was completely absent from SE samples and whose log2-fold expression was increased 1.5 fold or was completely absent from control samples were used to investigate over- and under-represented GO terms. Numbers of genes in each group are displayed in inset Venn diagram. (**a**) Biosynthetic and mRNA processes were over-represented in those genes only expressed or decreased in control SN sample. (**b**) Terms relating to biosynthetic, biological (conversely to control), and GPCR signalling were over-represented in the group of genes that were increased or exclusively bound after SE in SN compartment. (**c**) RNA processes, GPCR and glutamate signalling were over-represented in the genes that were exclusive to or decreased in control cytoplasm. (**d**) GO terms related to biological processes, biosynthetic processes, cell signalling, development (Dev.) and G protein coupled receptor (GPCR) signalling were over-represented by the genes that were exclusive to or increased after SE in cytoplasm. There was an under-representation of genes associated with responses to a stimulus in all groups and genes related with immune response were also under-represented in both the SN and cytoplasm fractions for genes exclusive to or decreased in controls.

Next, we investigated GO terms associated with transcripts that were expressed only in control samples or with a log2 fold-change ≤−1.5 after status epilepticus and exclusively in status epilepticus or with a log2 fold-change ≥1.5 after status epilepticus in SNs and cytoplasm (Fig. 4a–d and see Supplementary tables S5–S9). GO terms related to immune and stimulus-induced responses were under-represented and GPCR-signalling processes and/or pathways were over-represented in each group (Fig. 4a–d). Overall, there were more over-represented biological processes and pathways than under-represented ones after status epilepticus suggesting an increase in targeting of distinct class of transcripts after status epilepticus.

### RIP-seq identifies changes in the levels of Upf1-bound known and novel NMD transcripts

**Figure 5:**
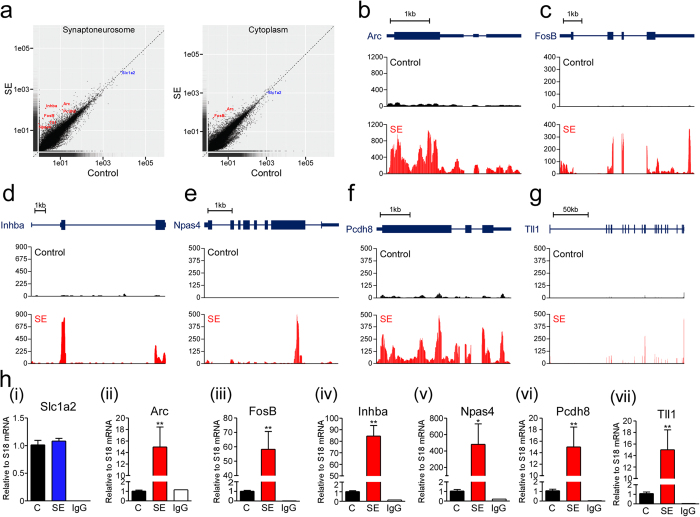
Validation of known and novel NMD targets identified by Upf1 RIP-seq. (**a**) Each transcript identified by Upf1 RIP-seq is marked by a black dot on the scatter plot. Transcripts that were upregulated after SE and were validated using PCR are marked in red. (**b-g**) The distribution of reads along each transcript in control and SE samples from synaptoneurosomes is presented. Note the markedly higher levels of reads found on the indicated transcripts after SE. (**h** i-vii) qPCR analysis of RNA isolated from ipsilateral hippocampus from control and SE mice validating RIP-seq data by showing a significant increase in Upf1-bound transcripts after SE. IgG was used as a negative control for IP (n = 5 per group; Mann-Whitney U-test C vs SE; *p < 0.05, **p < 0.01).

In order to look for specific transcripts that are targeted by Upf1 we explored the data for genes that were both significantly (p < 0.05) altered after status epilepticus and had a FPKM log2 fold change ≥1.5 (Supplementary tables S10, S11). Comparison of levels of identified transcripts between control and status epilepticus samples in the SN and cytoplasm fractions showed that status epilepticus increased Upf1-binding to a total of 16 annotated transcripts and 8 unannotated transcripts in SNs, and 6 annotated and 2 annotated transcripts bound to Upf1 after status epilepticus in the cytoplasm. From these increased Upf1-bound transcripts we chose six for validation in addition to one control which did not change after status epilepticus. These are highlighted on the scatterplots in Fig. 5a. *Arc* was included as it is a well-characterised NMD target^12^. *FosB* and *Pcdh8* both contain an up-stream in frame stop codon and in addition to *Tll1* were previously found in other studies^47^,^48^ so are likely bona fide NMD-regulated transcripts. *Inhba* and *Npas4* were chosen as they have not previously been associated with NMD regulation^8^,^45^,^46^,^47^,^48^,^49^,^50^. The distribution of sequencing reads on these six transcripts shows the marked increase in reads for these six transcripts (Fig. 5b–g). For RT-qPCR validation we used extracted mRNA from Upf1 pull-downs from whole hippocampus protein lysates in order to obtain sufficient biological replicates. Real-time PCR analysis showed the expected higher levels of *Arc, FosB, Inhba, Npas4, Pchd8* and *Tll1* bound to Upf1 after status epilepticus whereas levels of *Slc1a2* were not different between groups (Fig. 5h).

### Effects of small molecule NMD inhibitor on development of epilepsy after status epilepticus

**Figure 6:**
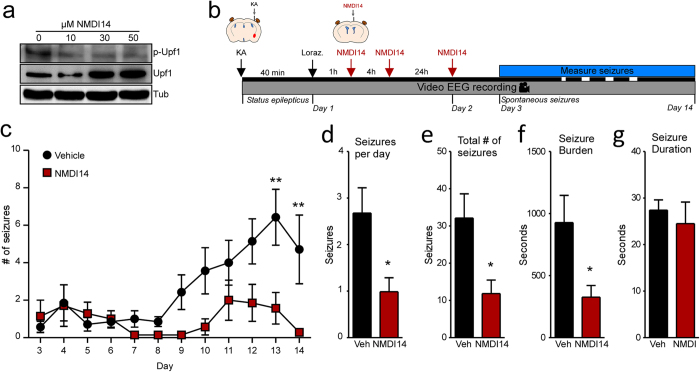
Inhibitor of Upf1, NMDI14, reduces spontaneous seizures after status epilepticus. (**a**) Representative western blot showing dose-range finding study on NMDI14 treatment that reduces levels of p-Upf1 in mouse hippocampus. Representative blots have been cropped to reduce unnecessary area. (**b**) SE was induced in mice by intraamygdala KA and lorazepam (Loraz) was administered after 40 min to curtail seizures and brain injury. Then, 1, 4 and 24 h later NMDI14 was administered ICV to achieve a final ventricular concentration of 50 μM. Spontaneous seizures were counted from day 3 to day 14 after SE using continuous video-EEG monitoring of mice. Original drawing by author Claire Mooney. (**c**) Graph showing spontaneous seizures recorded in each group. NMDI14 significantly reduced the number of seizures in mice compared to vehicle controls (n = 7/group; 2-way ANOVA with Bonferroni Post-hoc tests **p < 0.01). NMDI14 also reduced the total numbers of seizures had by mice for the duration of the study (**d**) and the total time spent having seizures (**e**)(n = 7/group, students t-test *p < 0.05). (**f**) There was no difference in the average duration of seizures in NMDI14 and vehicle control mice (n = 7/group, Student’s t-test p = 0.5854).

It is unknown whether the changes in transcript targeting by NMD as a result of status epilepticus affect functional outcomes. To investigate this, we targeted NMD after status epilepticus using a recently described NMD inhibitor, NMDI14^32^. Intraventricular administration of NMDI14 (10, 30 and 50 μM) reduced levels of p-Upf1 in the mouse hippocampus consistent with a reduction in NMD activity (Fig. 6a). No adverse behavioural effects were noted in any of the mice. We next investigated the effect of NMDI14 on the emergence and phenotype of epilepsy in the model. Mice underwent status epilepticus and were assigned to vehicle or NMDI14 treatment and were video-EEG monitored. NMDI14 was administered intraventricularly at 1, 4 and 24 hours after seizure termination by lorazepam and monitoring performed for 14 days with analysis beginning on the day of first spontaneous seizure (day 3 after status epilepticus) (Fig. 6b).

Spontaneous seizures emerged in both groups of mice within a few days of status epilepticus and NMDI14 had no effect on the emergence or time to first spontaneous seizure. However, NMDI14-treated mice had fewer spontaneous seizures overall during long-term monitoring compared to vehicle-treated mice (Fig. 6c–e). Total seizures and the daily seizure rate were both reduced (Fig. 6c,d) as was the overall seizure burden and the cumulative time in seizures in NMDI14-treated mice (Fig. 6d; P = 0.027). The main effect appeared to be during the later days of monitoring where acceleration of seizure frequency is normally observed in this model. The duration of individual seizures, when they occurred, was not different between vehicle- and NMDI14-treated groups (Fig. 6e; P = 0.5854).

## Discussion

RNA surveillance mechanisms such as the NMD pathway are increasingly recognized as important in nervous system development and function, synaptic plasticity and neurological diseases including certain epilepsies^19^,^20^,^21^,^22^,^23^. The present study found that status epilepticus produces changes to hippocampal levels of NMD proteins, including levels of Upf1, which is required for formation of mRNA surveillance complexes. These changes may reflect increased NMD activity and enhanced NMD capacity within the hippocampus that are adaptations to the increased transcription and perhaps higher rate of aberrant transcripts or transcripts expressing PTC or other sequences that engage the NMD pathway that accompany status epilepticus. Responses of the NMD machinery have not previously been investigated in status epilepticus although work had indirectly suggested changes to the NMD pathway occur after status epilepticus based on altered levels of known NMD-targeted mRNA transcripts and alternatively spliced versions of *Scn9a, Cdk5rap2* and *Stx2*^12^,^51^. Altered Upf1 levels were also found here in the hippocampus of patients with intractable epilepsy. Interestingly, we observed a tendency toward higher Upf1 levels in relation to hippocampal pathology although a larger sample size and details of pre-surgical seizure frequency will be required to validate this observation. Regardless, this indicates changes to NMD function may be present in chronic states of hyperexcitability and could be a potential target for the treatment of seizures in patients. The present studies did not explore the mechanism of increased Upf1 expression. Upf1 levels are known to be regulated by post-transcriptional mechanisms. Notably, microRNA-128 has been shown to target components of the NMD system, including Upf1^52^, and miR-128 levels are decreased in experimental and human epilepsy^53^.

NMD components and activity have been reported to localize to distal sites in neurons including axons where they locally regulate transcripts and specific splice variants in synaptic development and plasticity^13^. In addition to the overall increase in levels of select NMD components, we observed an increase in Upf1 levels within the synaptodendritic compartment after status epilepticus. This suggests accumulation or shuttling of NMD components to synapses and dendrites may follow episodes of strong or repetitive neuronal activity. This is consistent with the known importance of NMD activity in acting locally to limit the translation of transcripts such as *Arc* at synapses to maintain synaptic homeostatic mechanisms^12^,^54^,^55^. The increased co-location of Upf1 in the synaptodendritic compartment may enhance NMD capabilities to tightly regulate the levels of highly over-expressed activity-dependent transcripts such as *Arc*, limiting translation to protein. Consistent with this, we detected increased *Arc* levels bound to Upf1 after status epilepticus in the model. Together, these observations indicate that both prolonged acute seizures and perhaps chronic epilepsy alter NMD component levels and localization that may reflect homeostatic or mal-adaptive changes in the hippocampus to the transcriptional and translational burden imposed by seizures.

The present study provides the first analysis of Upf1-bound RNA transcripts in the hippocampus of mice subject to status epilepticus. Here we used Upf1 pull-down followed by RNA sequencing to identify and assess the abundance of mRNAs targeted by NMD. This has the advantage of providing much broader insights into the transcript landscape targeted by NMD and allows for the detection of unannotated and novel transcripts compared to techniques such as microarrays^8^,^45^,^46^,^47^,^48^,^49^,^50^. An additional technical advantage of our approach was to separately perform RIP-seq from different cellular compartments, enabling insight into functionally segregated regions of neurons where NMD targets may differ before and after status epilepticus. Our analyses found high transcript reads associated with Upf1 in the control mouse hippocampus as well as after status epilepticus, indicating NMD surveillance is active in the hippocampus even under basal conditions. Of note, reads were strongly enriched for the 3′UTR region of transcripts in all samples, accounting for between 35.1–38.7%. This compares to just 8% for another RNA binding protein, TDP-43^44^. This suggests there is substantial Upf1 enrichment on 3′UTRs expressed in the hippocampus, consistent with reports in other tissues^43^,^49^. Thus, NMD components may display preferences for specific features of transcripts, although this does not appear to change noticeably after status epilepticus. However, we did not sequence total input RNA for comparison and cannot draw stronger conclusions on whether Upf1-precipitated transcripts include longer 3′UTR domains. Also, our study did not explore whether poly-A transcripts were specifically enriched or quantify the relative coding versus noncoding species bound to Upf1 and whether this differed between cytoplasmic and synaptoneurosome fractions. These questions could be addressed in future studies via additional sequencing and by a modified experimental design.

Our GO analysis of Upf1 targeted transcripts in control samples indicates there is a set of biological processes which are specifically NMD targets including mRNA transport, transcription and protein processing. This fits with the restricted and compartmentalized location of NMD components in the hippocampus. Of note, numerous GO terms related to adhesion and development were over-represented in genes exclusive to SNs which is consistent with roles of NMD in growth and development of neurons^13^,^15^, processes also altered by status epilepticus^56^. Despite the known role of NMD in targeting immediate early gene transcripts such as *Arc*, GO terms related to responses to stimulus were under-represented. Also under-represented were terms associated with immune responses. This was surprising because status epilepticus is known to provoke a strong neuroinflammatory response and the NMD pathway is known to target transcripts with roles in immune responses^57^. We also did not observe particular enrichment of transcripts in specific neurotransmission pathways (e.g. cholinergic or glutamatergic), even in the synaptoneurosome fraction. The under-representation of such processes in our data may reflect the model or the timing of the analysis or indicate that these transcripts are regulated by other NMD core components or RNA surveillance mechanisms^1^,^4^.

The present study included validation of RIP-seq data that confirmed status epilepticus results in increased binding of numerous mRNA transcripts to Upf1. This included *Arc, FosB, Gadd45b, Pcdh8* and *Tll1* which have been previously identified as NMD mRNA targets^8^,^12^,^45^,^47^,^48^. Thus, our approach detected known and seizure-regulated targets of NMD pathway. The present study also identified changes to Upf1-bound transcripts that may be novel targets of the NMD; *Inhba* and *Npas4*. As for several other Upf1-bound transcripts, expression of both genes has been reported to be enhanced by seizures^58^,^59^,^60^. The function of these genes is not fully understood. Both transcripts were among several genes that are mediators of synaptic activity-induced acquired neuroprotection^61^ and other studies have suggested neuroprotective roles for Npas4 and Inhba^58^,^59^,^62^,^63^. Npas4 has also been reported to be required for the development and/or maintenance of inhibitory synapses and Npas4 knockout mice are prone to seizures^64^. Additional studies will be required to understand the mechanisms by which these transcripts fall under NMD regulation and whether their targeting by NMD serves a functional role in the outcome of status epilepticus. Some NMD targets we expected to find differentially expressed in our RIP-seq study were absent. This includes, C/EBP Homologous Protein (CHOP)^8^,^43^ which is upregulated by status epilepticus in the same model used here and protects against epilepsy development^35^.

Current treatments for epilepsy patients work through a limited number of mechanisms, including inhibiting various ion channels and neurotransmitter systems^65^. None are disease-modifying and there is no treatment to lower risk of seizures in someone who has experienced an epilepsy-provoking insult to the brain. The present study included a functional assessment of the contribution of NMD to the emergence of epilepsy after status epilepticus. Given the wide spectrum of transcript changes after status epilepticus it was difficult to predict whether interference with NMD would produce beneficial or detrimental effects. However, loss of microRNA-128, which negatively controls levels of Upf1 and another NMD protein^52^, results in seizures and premature death in mice^66^, suggesting that over-active NMD may contribute to the generation of seizures. We found that a small molecule NMD inhibitor, NMDI14, reduced the severity of the emergent epilepsy after status epilepticus triggered by intraamygdala KA. NMDI14 did not affect whether or not animals developed epilepsy but instead the treatment prevented the increase in frequency of spontaneous seizures in the model. This suggests that NMD activity may contribute most to progressive aspects of epilepsy in this model. This is surprising and contrasts with the protective role of Upf1 in models of neurodegeneration^67^. The cell and molecular cause(s) of seizure progression in the model are unknown so we do not know the mechanism by which NMDI14 reduced spontaneous seizures. Since secondary acceleration of seizures was affected this implies mechanisms other than those contributing to epileptogenesis per se. The best understood influence on seizure frequency in this model is neuroprotection^35^,^68^ but NMDI14 is unlikely to work through this mechanism since it was delivered after the initial insult. Rather, NMDI14 may influence processes such as synaptic reorganisation, neuroinflammation, gliosis or expression of neurotransmitter receptors^12^,^52^,^57^. Identifying the mechanism by which NMDI14 produces the observed effects could lead to the identification of novel targets for disease modification in epilepsy.

### Conclusion. 

The present study provides evidence for changes in the expression and activity of NMD in response to status epilepticus and indicates this system of RNA surveillance may also be altered in patients with epilepsy. We show initial evidence that targeting the NMD system can reduce the occurrence of spontaneous seizures. Whether targeting the NMD system represents a realistic target for the prevention of seizures or disease-modification in human epilepsy is uncertain. The NMD system is critical for normal neuronal function by maintaining strict regulation of the levels of aberrant transcripts. However, results here suggest a short period of treatment following an insult to the brain could be sufficient to yield disease-modifying effects while avoiding any potential detrimental effects of protracted inhibition. It will be important in future studies to investigate optimal dosing of this or similar NMD inhibitor compounds and validate disease-modifying effects in other models of acquired epilepsy.

## Additional Information

**How to cite this article:** Mooney, C. M. *et al*. RNA sequencing of synaptic and cytoplasmic Upf1-bound transcripts supports contribution of nonsense-mediated decay to epileptogenesis. *Sci. Rep.* **7**, 41517; doi: 10.1038/srep41517 (2017).

**Publisher's note:** Springer Nature remains neutral with regard to jurisdictional claims in published maps and institutional affiliations.

## Supplementary Material

Supplementary Figures

Supplementary Datasets
