# Supplementary material for: RNA sequencing of synaptic and cytoplasmic Upf1-bound transcripts supports contribution of nonsense-mediated decay to epileptogenesis
Source: Sci Rep. 2017 Jan 27;7:41517. doi: 10.1038/srep41517 (PMC5269742; doi:10.1038/srep41517)
Supplement: Supplementary Figures [file srep41517-s1.pdf]

Supplementary data figures

RNA sequencing of synaptic and cytoplasmic Upf1-bound transcripts supports contribution of nonsense-mediated decay to epileptogenesis

Claire M. Mooney, Eva. M. Jimenez-Mateos, Tobias Engel, Catherine Mooney, Mairead Diviney, Morten T. Venø, Jørgen Kjems, Michael Farrell, Donncha O'Brien, Norman Delanty, David C. Henshall.

Supplementary Data Figure S1

Simulated autopsy control experiment to assess effects of postmortem delay on Upf1.

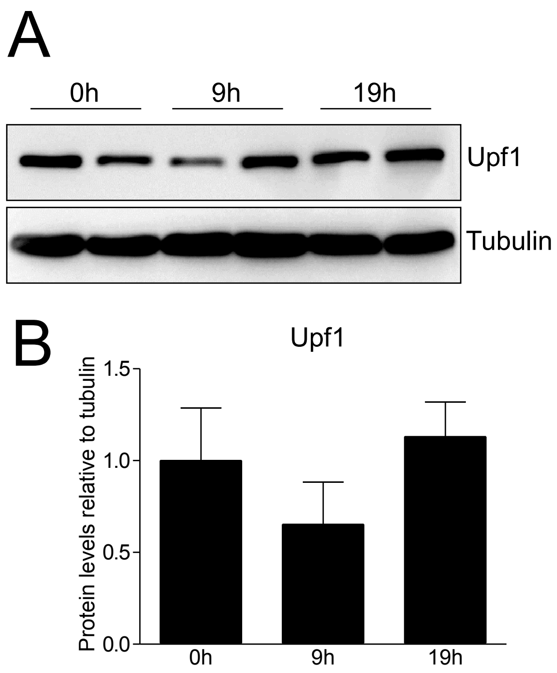

Figure S1

(A-B) As a control for the delay in time to autopsy for human samples naive mice were euthanised by cervical dislocation and left at 4°C for either 0h, 9h or 19h. Hippocampi were removed and protein was extracted to allow measurement of Upf1 levels by western blotting. No major difference in Upf1 levels was observed at any time point.

Supplementary data Figure S2

Comparison of under- and over-represented GO terms associated with genes identified in the present study and previously identified NMD targets.

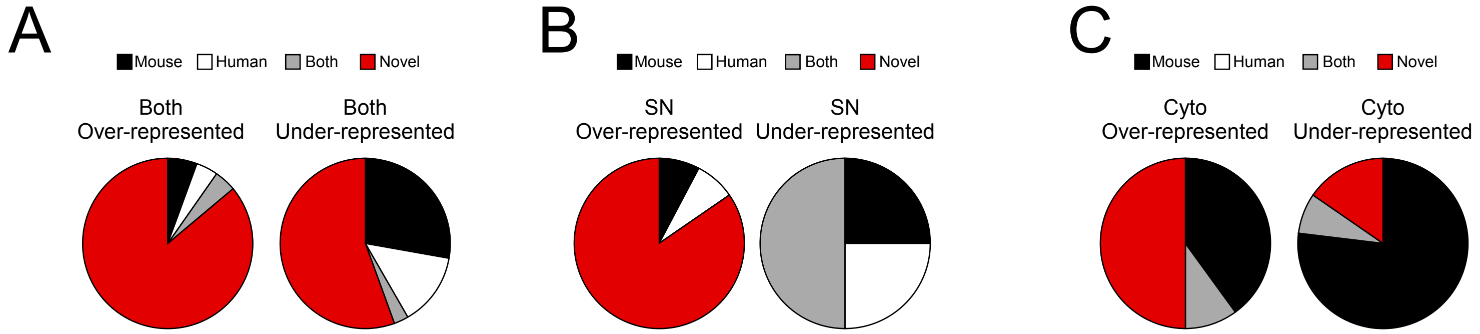

Figure S2

(A-C) A large proportion of the GO terms associated with genes identified in the present study are novel. Panther was used to generate a list of GO terms that are associated with genes that did not change in response to SE. These terms were compared to lists of GO terms generated using NMD targets identified previously in other studies in mouse and human tissue (Hurt et al 2013; Brazao et al 2012; Tani et al 2012; Maekawa et al. 2015; Mendell et al. 2004).

(A) Comparison of GO terms associated with genes that were unregulated in both synaptoneurosomes (SN) and cytoplasm (Cyto) fractions with terms associated with previously identified targets indicated that most of the over-represented and under-represented GO terms associated with data from this study were novel ones.

(B) Most of the over-represented GO terms associated with unregulated genes found exclusively in the SN fraction were novel to this study. No novel under-represented GO terms were identified in this study.

(C) Half of the over-represented GO terms associated with unregulated genes found exclusively in the Cyto fraction were novel to this study. ~15% (2/13) under-represented GO terms were novel to this study compared to previously published data.
